# Emerging strategies in lymph node-targeted nano-delivery systems for tumor immunotherapy

**DOI:** 10.1042/EBC20253008

**Published:** 2025-03-28

**Authors:** Yaoli Zhao, Muzi Tian, Xin Tong, Xiangliang Yang, Lu Gan, Tuying Yong

**Affiliations:** 1 National Engineering Research Center for Nanomedicine, College of Life Science and Technology, Huazhong University of Science and Technology, Wuhan 430074, China; 2 Key Laboratory of Molecular Biophysics of the Ministry of Education, College of Life Science and Technology, Huazhong University of Science and Technology, Wuhan 430074, China; 3 Hubei Key Laboratory of Bioinorganic Chemistry and Materia Medica, Huazhong University of Science and Technology, Wuhan 430074, China

**Keywords:** drug therapy, immune response, lymph nodes, nanoparticles, tumor microenvironments

## Abstract

The emergence of immunotherapy has led to the clinical approval of several related drugs. However, their efficacy against solid tumors remains limited. As the hub of immune activation, lymph nodes (LNs) play a critical role in tumor immunotherapy by initiating and amplifying immune responses. Nevertheless, the intricate physiological structure and barriers within LNs, combined with the immunosuppressive microenvironment induced by tumor cells, significantly impede the therapeutic efficacy of immunotherapy. Engineered nanoparticles (NPs) have shown great potential in overcoming these challenges by facilitating targeted drug transport to LNs and directly or indirectly activating T cells. This review systematically examines the structural features of LNs, key factors influencing the targeting efficiency of NPs, and current strategies for remodeling the immunosuppressive microenvironment of LNs. Additionally, it discusses future opportunities for optimizing NPs to enhance tumor immunotherapy, addressing challenges in clinical translation and safety evaluation.

## Introduction

Malignant tumors remain a leading global cause of death, with nearly 20 million new cases and 9.7 million cancer-related deaths reported in 2022. By 2050, new cases are projected to exceed 35 million [1], highlighting the urgent need for more effective cancer therapies to address this growing health crisis. Currently, various tumor immunotherapeutic drugs, such as immune checkpoint inhibitors, chimeric antigen receptor T-cell (CAR-T) therapies, and therapeutic tumor vaccines, have been approved for clinical use, achieving limited yet notable success [2]. These therapies primarily rely on activating T cells to amplify immune responses. However, the clinical response rate for solid tumors remains low at only 10–30% [3], largely due to insufficient T-cell activation, limited numbers of functional T cells, and impaired T-cell functionality in the tumor microenvironment [2,4]. Enhancing T-cell activation, expansion, and functionality remains a critical challenge in improving the efficacy of tumor immunotherapy.

Lymph nodes (LNs) serve as central hubs of immune responses, housing a large population of naive T cells that can be activated upon stimulation by tumor antigens. These T cells are crucial for anti-tumor immunity, as they specifically target and kill tumor cells. Structurally, LNs are encapsulated by a thin fibrous capsule and divided into organized compartments where T cells, B cells, and antigen-presenting cells (APCs) interact [5]. Targeted delivery of tumor antigens, immune adjuvants, or therapeutic agents to specific LNs regions enhances T-cell activation by promoting interactions between antigens and immune cells or between different immune cell types [6,7].

However, tumor cells and their secreted factors, such as lactic acid and extracellular vesicles (EVs), can metastasize to tumor-draining LNs (TDLNs), suppressing T-cell activation and promoting T-cell exhaustion, dysfunction, or regulatory T-cells (Tregs) differentiation. These factors may also alter stromal cell functions, further impairing anti-tumor immunity [8–10]. Consequently, remodeling the immunosuppressive microenvironment of TDLNs is essential for restoring T-cell activity and enhancing immunotherapy efficacy.

To address these challenges, targeting delivery by nanoparticles (NPs) has emerged as a promising solution. Compared with free drugs, NPs enable precise targeting of LNs, efficient delivery of therapeutic agents, and enhanced control over drug-release profiles. These systems offer unique advantages, including ease of surface modification, high tissue permeability, and excellent biocompatibility, making them ideal for both targeting LNs and modulating their microenvironment [11–14]. Furthermore, NPs facilitate the delivery of antigens, adjuvants, or immunomodulators to TDLNs, improving T-cell activation and reprogramming the immune microenvironment (IME) to support anti-tumor immunity.

This review highlights the structure and immune role of LNs, the impact of TDLNs immunosuppressive microenvironments, and strategies for designing NPs to enhance LNs targeting and functionality. It also discusses recent advancements, challenges, and future directions in LN-targeted NPs for tumor immunotherapy.

## Structure of LNs restricts targeting efficiency of NPs

### Structure of LNs

The lymphatic system plays a vital role in immunity, with LNs serving as central hubs for T-cell activation. Within their highly organized structure, LNs facilitate interactions between antigens and immune cells, driving T-cell activation and subsequent immune responses. Understanding LNs structure is essential for designing NPs to enhance targeted drug delivery and optimize immunotherapy outcomes.

LNs have a bean-shaped structure, with their outermost layer encased in a thin connective tissue capsule [15]. This connective tissue extends inward to form trabeculae, which divide the LNs into distinct regions. The barrier beneath the capsule consists of macrophages and lymphatic endothelial cells (LECs), and the area between these cells is referred to as the subcapsular sinus (SCS) [16].

The parenchyma of LNs is divided into cortical and medullary regions, with the cortex further subdivided into the superficial cortex and paracortex [17,18]. The superficial cortex, located below the SCS, contains spherical lymphoid follicles that are rich in B cells and follicular dendritic cells (FDCs). In contrast, the paracortex, situated between the cortex and medulla, is populated with abundant T cells and dendritic cells (DCs) [19]. High endothelial venules (HEVs), located in the paracortex, are specialized postcapillary venules composed of endothelial cells with a unique morphology. These endothelial cells provide sufficient intercellular spaces, enabling lymphocytes to migrate from the bloodstream into the LNs parenchyma and localize within the appropriate areas [20].

In the medulla, a large numbers of plasma cells, macrophages, and B cells are densely arranged to form medullary cords [6]. The medullary sinus, which lies between the medullary cords or between the cords and trabeculae, is rich in macrophages with antigen-capturing and scavenging functions [21]. The medullary sinus connects with the SCS, peritrabecular sinus, and both afferent and efferent lymphatics, ensuring seamless communication within the LNs structure.

The complex compartmentalization of LNs requires a robust skeletal framework to maintain its physical structure. Fibroblastic reticular cells (FRCs) in T-cell zones and FDCs in B-cell zones form this supportive network [22]. FRCs, along with the extracellular matrix (ECM) they produce, create conduits that regulate DCs and T-cell migration, residency, and function [22–24].

### Pathways for NPs entering LNs

The complex structure of LNs not only ensures efficient immune cell interactions but also creates unique physiological barriers that regulate the entry and distribution of therapeutic agents. For NPs, navigating these structural and cellular barriers is essential for achieving precise lymphatic targeting and maximizing therapeutic efficacy. Further, understanding the mechanisms by which NPs are transported into and within LNs is crucial.

NPs can enter LNs through two primary pathways. The first pathway relies on passive drainage, which is highly dependent on molecular weight and particle size. Small NPs (<70 kDa or <5.5 nm) can be directly transported from the SCS to B-cell or T-cell zones via conduits, where they are subsequently presented to T cells after being internalized by resident DCs [25–27]. In contrast, larger NPs, which are restricted by physical barriers such as the plasmalemma vesicle-associated protein secreted by LECs in the SCS (≥70 kDa or ≥5.5 nm), are instead captured by macrophages and DCs and translocated into the LNs parenchyma [28–30]. The second pathway for NPs’ entry into LNs depends on the CC-chemokine receptor 7-CC-chemokine ligand 19/21 (CCR7-CCL19/CCL21) axis. In this mechanism, antigens are recognized and captured by APCs in peripheral tissues, which induces increased expression of CCR7 on their surfaces [31]. LECs and FRCs in T-cell zones produce CCL21 and CCL19, while follicular DCs in B cell zones secrete CXC-chemokine ligand 13 (CXCL13) [20,32]. Additionally, LECs located at the top of the SCS express CC-chemokine ligand 1 (CCRL1), an atypical chemokine receptor that binds to CCL21. This binding creates a localized CCL21 concentration gradient, facilitating APCs migration from the SCS into the LNs parenchyma, where they present antigens to T cells [33,34]. Through this process, NPs leverage the homing properties of APCs to reach LNs. Interestingly, recent studies have identified a third pathway for biomolecule migration into LNs. SCS-lining LECs have been observed to translocate large molecules (up to 500 kDa) into T-cell zones via fluid-phase transcytosis. This mechanism highlights an alternative route for large biomolecules to access the LNs parenchyma [35].

In conclusion, the rational design of NPs, guided by the physiological structure of LNs to effectively navigate or bypass barriers and achieve precise drug accumulation, holds great potential for further improving the efficacy of tumor therapy.

## Factors affecting LNs targeting efficiency of NPs

### Administration routes

The route of administration plays a pivotal role in determining the *in vivo* behavior of NPs and their efficiency in targeting LNs. Four main delivery methods are utilized for lymphatic targeting: intranodal injection, interstitial injection, intravenous injection, and oral administration (Table 1).

**Table 1:** Summary of the characteristics and limitations of different administration routes for LNs targeting.

Administration route	Characteristics	Limitations	Ref.
Intranodal (I.LN)	Direct and efficient LN targeting Low drug doses needed	Technically challenging Difficult LN localization Risk of tissue damage	[36–41]
Interstitial (I.D., S.C., I.M.)	**I.D**.: Efficient LNs delivery via initial lymphatics/HEVs **S.C**.: Indirect flow into lymphatics with lower efficiency than I.D. **I.M**.: Effective T-cell activation in TDLNs	Efficiency depends on lymphatic flow S.C. delivery has lower bioavailability I.M. requires site-specific studies	[42–46]
Intravenous (I.V.)	Widely used in clinical practice Modifications can extend NPs circulation time	Clearance by mononuclear phagocyte system Accumulate in liver or kidneys Poor LNs targeting	[46,47]
Oral (P.O.)	Most convenient for clinical uses Mesenteric LNs-targeted delivery Effective for lipophilic particles	Low stability in acidic gastrointestinal Intestinal mucus and epithelial barriers Poor bioavailability	[47–49]

LN, lymph node. NPs, nanoparticles. HEVs, high endothelial venules. TDLNs, tumor-draining LNs.

Intranodal injection is the most direct and effective approach, as it delivers low doses of drugs directly into LNs, eliciting robust immune responses [36,37]. However, its practical application is highly restricted due to technical challenges, including the complexity of the injection process, difficulty in LNs localization, and the potential risk of tissue damage [38–40]. Interstitial injection, a more commonly adopted method in clinical practice, includes subcutaneous (S.C.), intradermal (I.D.), and intramuscular (I.M.) delivery [42,43]. Among these, I.D. injection facilitates drug transport into LNs via initial lymphatics under the epidermis or through HEVs. In contrast, S.C. injection primarily relies on indirect flow into lymphatic vessels, leading to lower bioavailability and delivery efficiency compared with I.D. administration [44,45]. I.M. injection, often used in vaccination, has shown efficacy in activating specific T cells in TDLNs when the appropriate muscle site is selected [48,50]. Intravenous administration, although widely used, faces challenges as NPs are absorbed by the mononuclear phagocyte system and accumulate in the liver or kidneys before entering interstitial tissues and lymphatic vessels [49]. While NPs modifications can extend circulation time, systemic administration remains limited by inefficient LNs targeting. Oral administration offers the greatest convenience for clinical applications. NPs can reach mesenteric LNs via two mechanisms: targeting M cells to enter lymphatic vessels or being transported through chylomicrons in M cells, which is particularly effective for lipophilic particles [41,49]. However, the acidic gastrointestinal environment, coupled with intestinal mucus and epithelial barriers, significantly reduces the bioavailability of NPs, posing challenges to therapeutic efficacy [46].

### Physical properties of NPs

Modulating the physical properties of NPs, including particle size, surface charge, hydrophilicity, and deformability, significantly influences their accumulation in LNs and enhances bioavailability.

#### Particle size

Particle size plays a critical role in determining the efficiency of LNs targeting. The flow rate in blood capillaries is 100 to 500 times higher than in lymphatic capillaries, resulting in rapid clearance of small NPs (<10 nm) following interstitial administration. However, the cellular gaps in lymphatic capillaries (30–120 nm) are considerably larger than those in blood capillaries (approximately 10 nm) [49]. Consequently, particles with diameters of 10–100 nm can passively enter lymphatic vessels, while larger particles (>100 nm) are restricted by the ECM pore size, leading to entrapment in the interstitial matrix [47,51–53].

**Figure 1:**
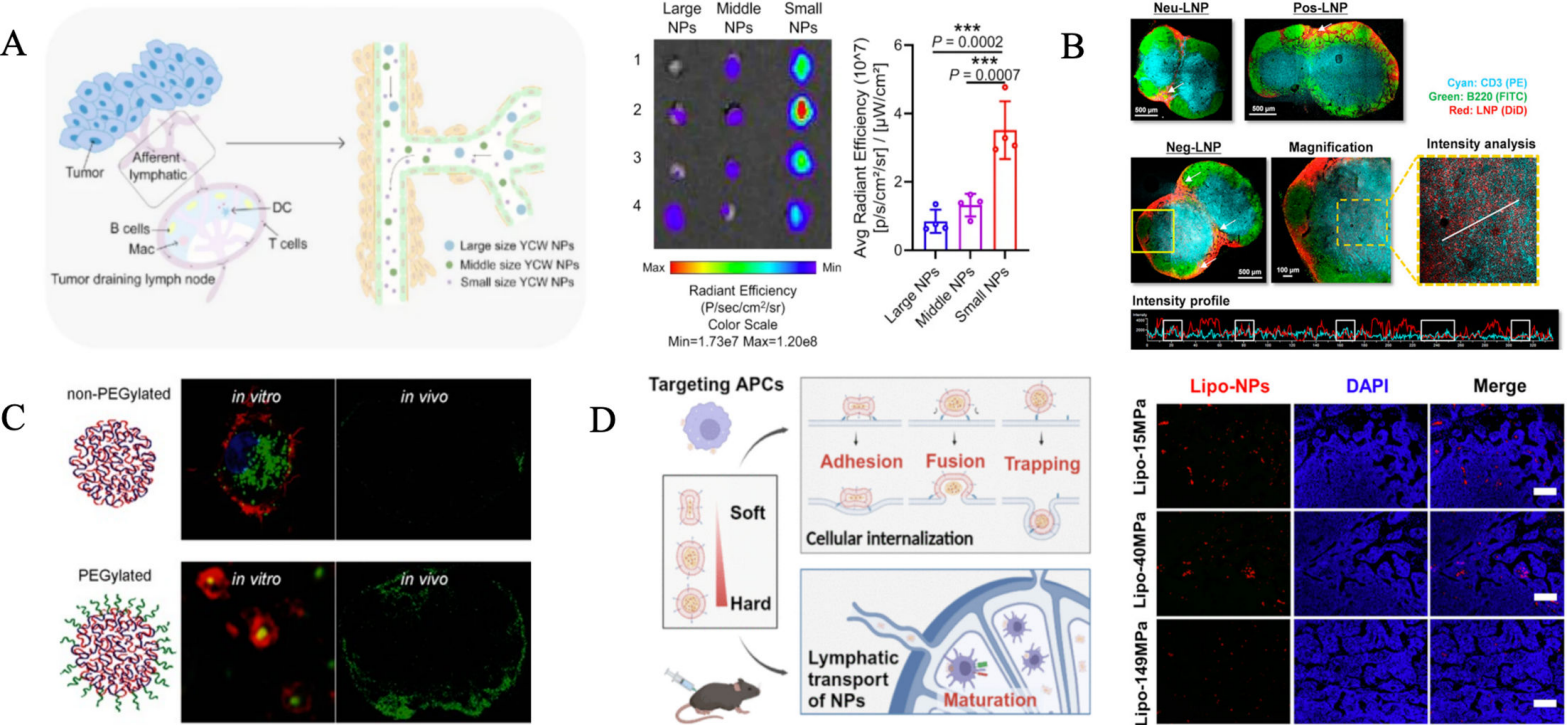
Influence of NP physical properties on LN targeting efficiency. (**A**) *Ex vivo* fluorescence images of LNs at 48-h post-injection of NPs with varying sizes. Copyright 2019 Springer Nature. (**B**) Fluorescence images of LNs following treatment with liposomes of different surface charges. Copyright 2020 American Chemical Society. (**C**) Fluorescence images of LNs demonstrating enhanced vaccine and drug delivery using PEGylated nanoparticles. Copyright 2016 John Wiley and Sons. (**D**) Immunofluorescent images of LN sections showing the distribution of liposomes with varying Young’s moduli. Copyright 2024 American Chemical Society. LNs, lymph nodes; NPs, nanoparticles; PEG, polyethylene glycol.

Xu et al. [54] conducted a comparative study on the distribution of NPs (50, 200, and 500 nm) in LNs 48 hours (h) after intratumoral injection. Their findings revealed that small NPs (50 nm) exhibited significantly higher accumulation in LNs compared with larger particles (Figure 1A). Similarly, Vania Manolova et al. [55] examined NPs’ transport in LNs after injecting various-sized particles into mice footpads. The results indicated that small NPs (20–200 nm) could directly penetrate LNs parenchyma via the SCS and accumulate in B-cell regions within 2 h, where they were captured by resident DCs. In contrast, large particles (500–2000 nm) relied on DCs’ uptake in peripheral tissues before entering the SCS, ultimately accumulating in the paracortex and medulla at around 8 h. Further advancements were demonstrated by Alex Schudel et al. [56], who developed a programmable multistage drug-delivery platform (NP-OND). This platform used NPs with biodegradable, programmable linkers on their surface, enabling rapid cargo release upon LNs targeting. Compared with free particles and compounds, the platform significantly improved targeting efficiency, allowing the cargo to interact with more lymphocyte subtypes and increasing targeting numbers by orders of magnitude. These findings underscore two key points: small NPs (10–100 nm) exhibit superior efficiency in targeting LNs due to their ability to directly access LNs parenchyma, whereas large NPs primarily rely on APC-mediated transport, a less efficient but viable pathway [38,40].

#### Surface charge

Surface charge is a critical determinant in the design of NPs targeting LNs. Positively charged NPs are rarely employed for LNs targeting due to several limitations. The ECM surrounding lymphatic vessels comprises structural proteins and glycosaminoglycans, forming a negatively charged, complex network [57]. When positively charged NPs encounter this environment, they are easily trapped within the ECM, significantly reducing transport efficiency. Additionally, positively charged NPs tend to form protein coronas *in vivo*, which may alter the structure and functionality of surface ligands [6,58,59]. More importantly, Kedmi et al. [60,61] and Goodman et al. observed that intravenously administered positively charged lipid NPs could induce liver toxicity and inflammatory responses, underscoring their potential safety concerns. Although positively charged NPs are more readily internalized by APCs compared with neutral or negatively charged particles, their disadvantages, including reduced delivery efficiency, impaired active targeting, and higher cytotoxicity, have largely restricted their applications [62].

In contrast, negatively charged or neutral NPs exhibit stronger LNs accumulation and enhanced targeting efficiency [63,64]. Nakamura et al. [65] conducted a study comparing the LNs transitivity of liposomes with varying surface charges (all with a uniform particle size of 30 nm) (Figure 1B). Their findings demonstrated that negatively charged liposomes outperformed neutral ones in targeting LNs, while positively charged liposomes exhibited significantly reduced transport efficiency due to their interactions with the ECM. Recently, charge-reversal materials have gained attention as a promising strategy for overcoming the limitations of surface charge. These materials enable charge conversion to enhance cellular uptake while minimizing environmental constraints [66–68]. For instance, Zhang et al. [69] designed a reactive oxygen species-responsive hydrogel loaded with doxorubicin and a nanoadjuvant. Upon intratumoral injection, doxorubicin induced immunogenetic cell death of tumor cells, leading to the *in situ* release of negatively charged antigens. The positively charged hydrogel then adsorbed antigens, resulting in charge reversal. This mechanism helped the hydrogel evade macrophage phagocytosis and facilitated its drainage to LNs. Ultimately, the enhanced LNs targeting activated DCs and improved the therapeutic effects.

#### Hydrophilicity/Hydrophobicity

After entering the human body, NPs are primarily dispersed in aqueous solutions. Hydrophilic NPs are more effectively transported through water channels, whereas hydrophobic NPs are more readily internalized by APCs [70,71]. Thus, achieving a balance between these two processes, efficient LN targeting and APC internalization, relies on the precise regulation of the hydrophilicity of NPs.

Polyethylene glycol (PEG) is a widely used hydrophilic molecule for NPs surface modification, which offers improved stability by reducing enzymatic degradation and clearance by the circulatory system. When appropriately optimized in terms of PEG ratio and chain length, PEGylated NPs can achieve efficient LN targeting [72–75]. De Koker et al. [76] developed PEG-modified PMA NPs (PEG-PMA) and found that PEGylation significantly improved LNs accumulation compared with non-PEGylated PMA NPs. This enhancement was attributed to reduced fouling behavior, as PEGylation blocked the residual free thiol and pyridyldisulfide groups on the NPs, thereby minimizing interactions with the ECM. As a result, PEGylated NPs more effectively reached LNs and targeted diverse immune cell subpopulations (Figure 1C).

#### Deformability

Mechanical properties, such as elasticity, play a critical role in influencing the biological behavior of NPs and their interactions with the immune systems [77]. Modulating the elasticity of NPs can significantly alter their cellular uptake efficiency and endocytosis pathways, though different immune cells respond to these mechanical changes in distinct ways [6,78].

Zou et al. [79] synthesized mesenchymal stem cell membrane-encapsulated silica NPs (MCSNs) with varying elasticities and observed that macrophage uptake was substantially lower for soft MCSNs compared with rigid ones. Conversely, Song et al. [80] demonstrated that deformable albumin-stabilized emulsions loaded with antigens enhanced antigen uptake by bone marrow DCs more effectively than their solid albumin counterparts. This suggests that elasticity-dependent effects on cellular uptake vary among immune cell types.

To further investigate the influence of elasticity on endocytosis pathways, Yuan et al. [81] developed NPs (Lipo-NPs) with adjustable elasticity by cross-linking different concentrations of Zn^2+^ ions with nucleic acids (5′-GMP) and co-encapsulating them with an immunoadjuvant in liposomes. Upon co-incubation with macrophages, soft Lipo-NPs adhered to the cell membrane before entering the cell, moderately hard Lipo-NPs utilized membrane fusion, and hard Lipo-NPs relied on lysosome-mediated classical phagocytosis. Additionally, Yuan et al. examined the accumulation of these Lipo-NPs in TDLNs after intratumoral injection. Their findings revealed that softer Lipo-NPs primarily relied on passive drainage to reach TDLNs, whereas stiffer Lipo-NPs depended on migratory APCs for translocation (Figure 1D). These results highlight that elasticity not only affects cellular uptake mechanisms but also shapes the interactions between NPs and the immune system.

### Surface modification of NPs

Surface modifications, including biological and chemical strategies, are critical for enhancing the functionality of NPs. By leveraging receptor–ligand interactions, these modifications significantly improve the precision and specificity of LNs targeting, enabling more efficient drug delivery and therapeutic outcomes.

#### Targeted ligands

A growing body of research has focused on engineering ligands on NPs to enhance their targeting efficiency toward LNs by interacting with APCs or LECs. These ligands enable NPs to activate resident APCs or ‘hitchhike’ on migratory APCs for transport into LNs.

**Figure 2:**
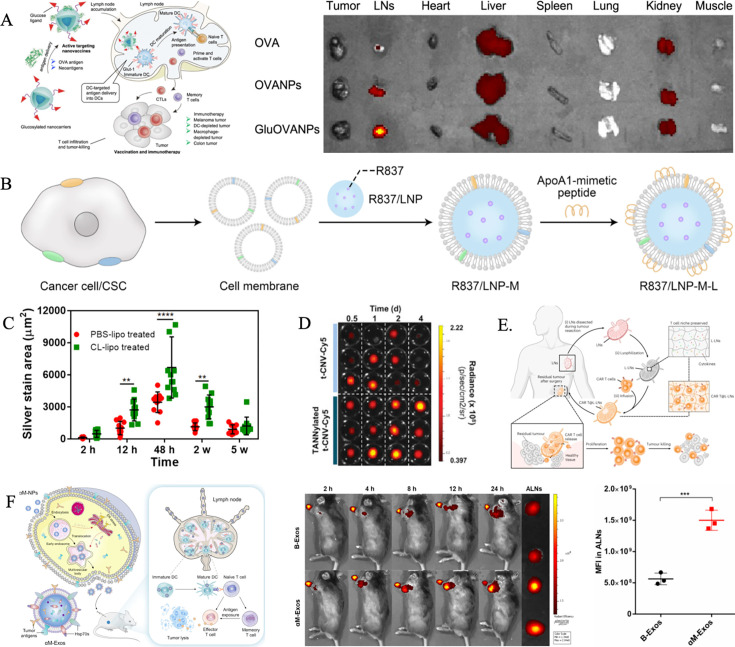
Influence of NP surface modification on LNs targeting efficiency. (**A**) Accumulation of glucosylated nanovaccines delivering neoantigen in LNs. Copyright 2024 American Chemical Society. (**B**) High transport efficiency into lymphatic vessels using HDL-mimicking liposomal vectors modified with the 22A peptide of apolipoprotein A1. Copyright 2024 American Association for the Advancement of Science. (**C**) Follicular accumulation of ovalbumin-conjugated spherical gold NPs following depletion of subcapsular sinus macrophages. Copyright 2020 American Chemical Society. (**D**) Fluorescence intensity of TDLNs treated with bioadhesive tannic acid-incorporated t-CNV. Copyright 2024 Wiley-VCH GmbH. (**E**) Implantation of freeze-dried, patient-derived LNs loaded with mesothelin-targeting chimeric antigen receptor T cells to promote cell proliferation and prevent tumor recurrence. Copyright 2024 Springer Nature. (**F**) LN targeting efficiency of αM-Exos. In LNs, αM-Exos were endocytosed by dendritic cells, triggering T cells to specifically target and kill tumor cells. Copyright 2024 American Chemical Society. HDL, high-density lipoproteins; LNs, lymph nodes; NPs, nanoparticles.

One example is glucose transporter protein 1, which is highly expressed on DCs due to their glucose-dependent metabolism. Liu et al. [82] exploited this feature by developing glucosylated nanovaccines for neoantigen delivery. These nanovaccines exhibited high LNs accumulation and were preferentially taken up by resident DCs following S.C. administration, leading to improved antigen delivery efficiency (Figure 2A). Similarly, ligands targeting CLEC9A [83], DEC-205 [84], and mannose receptors [85] have been used to enhance APC-mediated LN targeting. Additionally, antibodies such as CD11c [86] and CD11b [87] have been conjugated to NPs, facilitating their uptake and subsequent transfer to LNs. Besides APC targeting, NPs can also be designed to interact with lymphocytes and LECs. For instance, lymphocytes express scavenger receptor class B type I, which facilitates the transport of high-density lipoproteins (HDL) into lymphatic vessels. Leveraging this pathway, Liu et al. [63] developed HDL-mimicking liposomal vectors modified with the 22A peptide of apolipoprotein A1. These large, mildly negatively charged liposomes (50 nm, −5 mV) demonstrated significantly higher LNs accumulation and DCs uptake compared with smaller, highly negatively charged liposomes (40 nm, −30 mV) without peptide modification (Figure 2B). The results underscore the importance of active infiltration mechanisms in enhancing LNs targeting.

Beyond NPs’ modification, reforming LNs environments has also shown potential for improving targeting precision. Qin et al. [88] utilized a two-step click chemistry-based strategy to pre-modify LECs surfaces. First, azide-modified DSPE-PEG was injected subcutaneously to conjugate with albumin and translocate to the SCS, exposing azide groups on LECs surfaces. After 24 h, dibenzocyclooctyne-modified liposomes were reinjected, allowing selective binding to the azide groups. This approach enabled precise delivery of antigens and immune adjuvants to LECs, which were then efficiently absorbed by resident DCs.

The accumulation of NPs in LNs is influenced not only by their design to target APCs and LECs but also by SCS macrophages. These macrophages form the first line of immune defense by creating a barrier to LNs. While some studies have reported that SCS macrophages capture and deliver antigens and NPs to DCs, others suggest that depleting SCS macrophages can facilitate the propagation of tumor EVs into LNs, enhancing the immune response [89,90]. Zhang et al. [91] systematically investigated the role of SCS macrophages in NPs translocation. Their study demonstrated that preemptive depletion of SCS macrophages in mice through I.D. injection of clodronate liposomes significantly enhanced the delivery and accumulation of ovalbumin-conjugated spherical gold NPs (OVA-AuNPs) in LNs follicles. This strategy not only prolonged the retention time of the NPs but also increased the efficiency of antigen uptake and presentation by follicular DCs (Figure 2C). These findings highlight that selectively eliminating the LNs barrier created by SCS macrophages can enhance nanocarrier system efficacy by improving LNs targeting, antigen retention, and immune activation.

Moreover, active strategies to bypass complex physiological barriers and enable NPs to penetrate parenchymal tissues within LNs have proven to significantly enhance drug accumulation. Jin Seung Mo et al. [92] developed a drug delivery system named BIND, which conjugates protein antigens with an immune adjuvant (t-CNV) and incorporates bioadhesive tannic acid (TA). The phenolic hydroxyl groups in TA form strong bonds with elastin in the SCS and lymphatic vessels, allowing BIND to achieve efficient LNs’ accumulation and retention (Figure 2D). Upon exposure to proteases abundant in the SCS, t-CNV is gradually released from BIND, enabling its accumulation in the paracortex due to its small size and high elastin affinity. This targeted delivery through LNs conduits subsequently triggers specific immune responses. Tissue-mediated approaches further enhance delivery efficiency by utilizing natural tissue structures as reservoirs for drugs or cells, providing a promising avenue for tumor therapy. Shi et al. [93] demonstrated this by freeze-drying patient-derived LNs (that had not undergone radiation or chemotherapy) to create lyophilized LNs (L-LNs). These L-LNs retained their porous structure, along with cytokines and chemokines, despite having few surviving cells. They were loaded with mesothelin-targeting CAR-T cells and implanted into the tumor resection cavity in mice. The implanted LNs promoted cell proliferation, maintained a memory phenotype, and suppressed tumor recurrence (Figure 2E). This approach underscores the potential of tissue-mediated strategies for NPs delivery in enhancing therapeutic outcomes.

In conclusion, these studies demonstrate innovative modifications of LNs at cellular, structural, and organizational levels, providing diverse perspectives for achieving precise LNs targeting in nano-delivery platforms.

#### Biofilm coating on NPs

Encapsulating biofilms on NPs as drug carriers, rather than directly modifying ligands or antibodies, has emerged as a prominent research focus in targeting delivery. These biomimetic NPs offer excellent biocompatibility, prolonged circulation time, and functional integration through proteins on cell membranes [94]. Wang et al. [95] camouflaged NPs with mature DCs membranes, enabling the particles to inherit surface proteins such as major histocompatibility complex-1 (MHC-I), CD80, CD86, and CCR7. These biomimetic NPs demonstrated LNs homing capabilities, presenting tumor-associated antigens to T cells for effective activation. Similarly, Zhang et al. [96] used macrophage membranes to coat NPs, enhancing phagocytosis efficiency while maintaining macrophage viability. These particles targeted inguinal LNs, slowed antigen release, and induced a durable immune response. In another approach, Li et al. [97] wrapped NPs with tumor cell membranes, which facilitated migration to TDLNs and promoted T cell and natural killer cell activation through enhanced antigen presentation by DCs.

EVs, nanoscale lipid bilayer vesicles secreted by cells, represent another biomimetic strategy in NPs. EVs inherit surface proteins and intracellular contents from their parent cells, and their functionalization can be further enhanced through pre-incubation, genetic engineering, and other techniques. Liang et al. [1] created biologically self-assembled EVs (αM-Exos) by loading NPs with inducers to treat tumor cells. Upon subcutaneous administration, αM-Exos efficiently migrated to LNs, where they were endocytosed by DCs, triggering T cells to specifically target and kill tumor cells (Figure 2F). Tong et al. [98] developed a cancer vaccine by fusing tumor cell-derived EVs with cationic liposomes and bacteria-derived outer membrane vesicles. This vaccine successfully delivered tumor antigens and adjuvants to LNs via S.C. injection, promoting DCs maturation and T-cell activation. Additionally, Liang et al. [99] modified EVs with albumin-binding domains, which bound strongly to serum albumin after entering the body. This modification extended EVs circulation time, enhanced LNs accumulation, and boosted immunomodulation efficacy.

In conclusion, biofilm-coated NPs and EV-based strategies have shown great promise in enhancing targeted delivery, LNs accumulation, and immune activation, offering innovative solutions for effective cancer immunotherapy.

## Immunoregulatory properties of NPs in LNs

The IME consists of immune cells, stromal cells, and the ECM, which collectively maintain the balance between immune activation and tolerance within LNs. During inflammation, the immune response disrupts this balance to combat pathogens. However, as tumors progress, the emergence of ‘variable’ tumor cells can reshape the IME in LNs through various pathways, potentially affecting therapeutic efficacy. Therefore, understanding the composition and formation of the IME is crucial, as targeted modifications may enhance tumor therapy outcomes.

### Regulation of immunosuppressive cells by NPs

**Figure 3:**
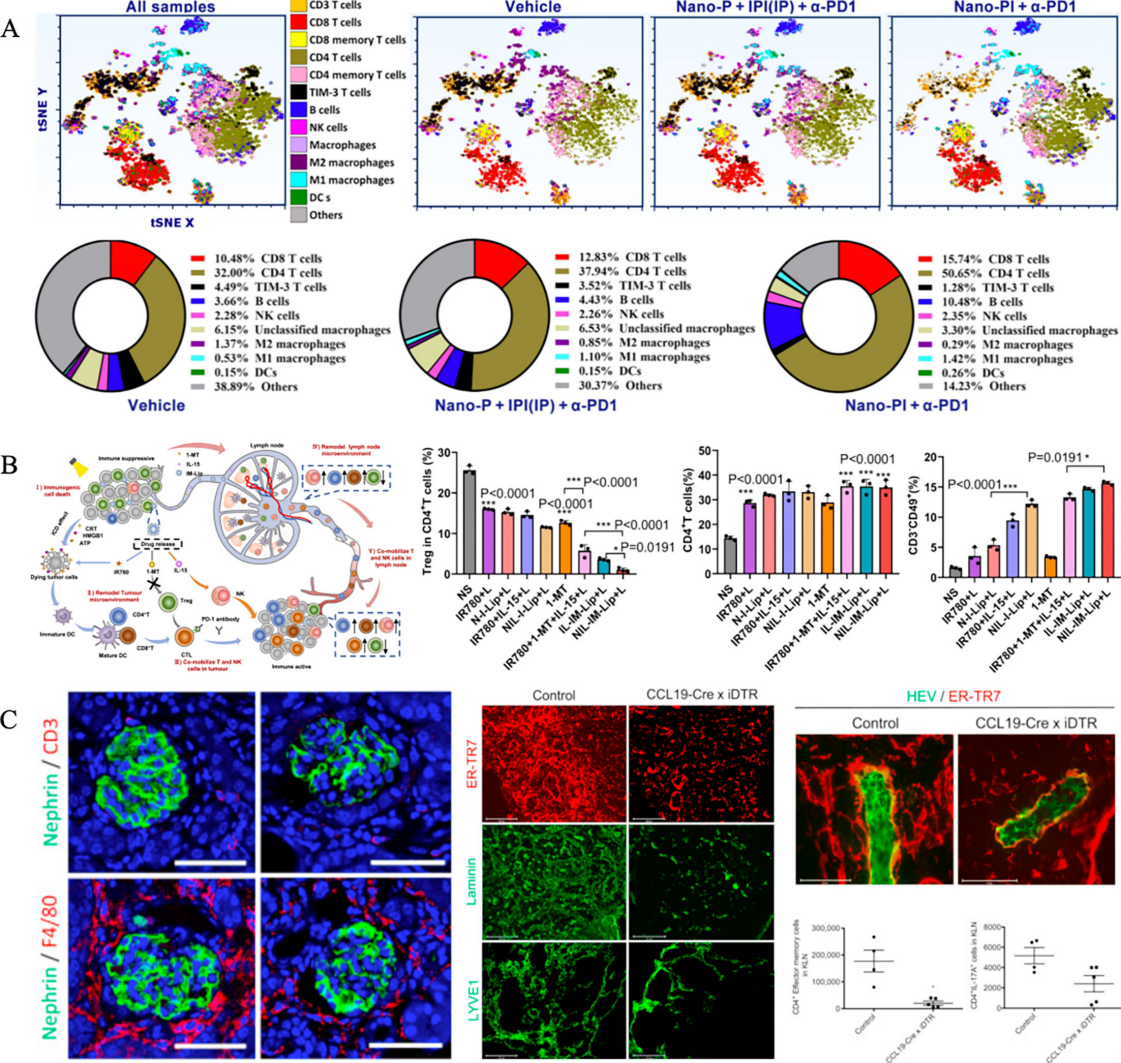
Immunoregulatory properties of NPs in LNs. (**A**) Iterations in immune cell subpopulations in LNs following treatment with albumin NPs co-loaded with immunomodulators and paclitaxel. Copyright 2022 American Association for the Advancement of Science. (**B**) Restoration of the LNs IME through Tregs inhibition and co-activation of T cells and NK cells by a pH- and temperature-sensitive liposome loaded with a photothermal agent and an IDO1 inhibitor, and modified with IL-15 and tumor-specific targeting ligands. Copyright 2023 Springer Nature. (**C**) Decreased fiber density and reduced high endothelial venule pocket formation in kidney LNs through FRC depletion. Copyright 2019 Elsevier. FRC, fibroblastic reticular cell; IME, immune microenvironment; LNs, lymph nodes; NPs, nanoparticles.

Tumor-associated macrophages are categorized as pro-inflammatory (M1) or anti-inflammatory (M2) macrophages [100]. M2 macrophages contribute to immunosuppressive environments by secreting factors that promote tumor growth and metastasis. Their infiltration into LNs can compromise the efficacy of cancer treatments. To address this, Song et al. [101] designed albumin NPs (Nano-PI) co-loaded with immunomodulators and paclitaxel. When combined with anti-programmed death-1 (α-PD1) therapy, Nano-PI effectively delivered its payload to both tumors and LNs, promoting M2-to-M1 polarization, increasing T cells and DCs populations, and Tregs (Figure 3A). This strategy successfully remodeled the IME at both sites. Similarly, Tong et al. [102] developed size-switchable NPs capable of targeting tumors and LNs simultaneously. In the acidic tumor microenvironment, the NPs cleaved, enhancing the component penetration into tumor and LNs parenchyma while reprogramming M2 macrophages to M1, thereby restoring anti-tumor immunity.

Tregs also play a significant role in establishing IME in both LNs and tumors. Their accumulation in LNs can inhibit the infiltration of activated T cells into tumor tissue. To counteract this, Fu et al. [103] developed a pH- and temperature-sensitive liposome loaded with a photothermal agent, an IDO1 inhibitor, and modified with interleukin-15 (IL-15) and tumor-specific targeting ligands. Upon reaching the tumor, the liposome cleaved and release IL-15 and IDO1 inhibitor while draining into LNs in a size-dependent manner. IL-15 enhanced the activation of T cells and natural killer cells, while IDO1 inhibitor suppressed Tregs, effectively restoring the IME in LNs and demonstrating potent anti-tumor activity (Figure 3B).

### ECM remodeling

FRCs are structural support cells that maintain the integrity of LNs architecture. In recent years, FRCs have been recognized for their immunoregulatory functions [104,105]. They secrete chemokines to mediate interactions between APCs and lymphocytes, and interleukin-7 (IL-7) to support T cells’ survival [106]. Additionally, FRCs produce nitric oxide, which inhibits T cells’ proliferation and functionality [107]. Beyond biochemical pathways, the cytoskeletal mechanics of FRCs’ cellular networks also play an essential role in immune responses [108]. For instance, during the initiation of immune responses, LNs expand and deform to accommodate the recruitment and activation of T cells and DCs. This process is mediated by FRCs stretching and proliferation [109].

Emerging evidence suggests that tumor cells metastasize by altering the number, phenotype, or mechanical properties of FRCs in TDLNs. Apollo et al. [110] analyzed transcriptome data from diffuse large B cell lymphoma patient samples and mouse models, revealing that FRCs in LNs undergo reprogramming and activation, which suppress T cells function. Similarly, Han et al. [8] demonstrated that in head and neck squamous cell carcinoma, FRCs engulf tumor-derived EVs and up-regulate PD-L1 expression, leading to CD8^+^ T-cell depletion and promoting the formation of an immunosuppressive LNs’ microenvironment. Riedel et al. [10] found that lactic acid from tumor cells diverts to LNs, disrupting mitochondrial function and transforming FRCs into cancer-associated fibroblast-like phenotypes. Rovera et al. [111] further showed that de-differentiated melanoma cells secrete interleukin-1 (IL-1), inhibiting FRCs contraction and promoting their relaxation, activation, and proliferation, thereby enhancing tumor cell invasion.

Targeting the biochemical or physical properties of pre-metastatic FRCs offers a potential strategy to improve LNs IME [104]. For example, a study [109] investigating crescentic glomerulonephritis used the CCL19-Cre×iDTR mouse models and diphtheria toxin to selectively deplete FRCs from kidney LNs. This intervention reduced CD4^+^ T-cell activation in kidney LNs, mitigating kidney damage (Figure 3C). This finding highlights the potential therapeutic value of regulating FRCs in immune responses. However, Onder et al. [112] noticed the critical role of FRCs in the formation of T-cell environments, including tertiary lymphoid structures and T-cell tracks at the tumor site. They found that ablation of FRCs precursors in tumor-bearing mice resulted in a significant reduction in T-cell-mediated anti-tumor activity, underscoring the essential role of FRCs in the immune responses.

In tumor immunotherapy, most current strategies primarily focus on modulating immune cells in LNs. However, stromal cells, such as FRCs, remain largely underexplored as potential targets for reshaping the IME. Integrating small-molecule drugs or NPs designed to either enhance or suppress FRCs functions could open new avenues for cancer treatment. To facilitate clinical translation, it is essential to further elucidate the heterogeneity of FRCs across different cancer types and stages, as well as to conduct comprehensive safety evaluations to ensure therapeutic efficacy and minimize adverse effects.

## Outlook

It has been demonstrated that adjusting size, surface charge, hydrophilicity, deformability, or surface modification maximizes the efficiency of NPs for LNs translocation and internalization by immune cells. On one hand, functionalized NPs directly stimulate T cells and avoid immune evasion. On the other hand, these materials indirectly remodel IME of LNs by reducing suppressive immune cells and reprogramming activated stromal cells to create a more suitable environment for T-cell activation. While these strategies have demonstrated efficacy in animal models, they remain in research stage and have not yet been implemented clinically because factors, such as the long-term safety in humans, the effect on immune network of LNs, and the actual delivery efficiency, still need to be further clarified. For example, L-BLP-25, a liposomal vaccines, could activate tumor antigen-specific anti-tumor immune response through delivering tumor antigen peptides into patients. Due to unexpected efficacy in a Phase III clinical trial, the program was terminated. Therefore, to facilitate better clinical translation, interdisciplinary approaches predicting the safety and validity of carriers ahead and well-established evaluation metrics may be beneficial. In addition, for industrialization, rational scalable manufacturing processes, appropriate storage methods and strict regulatory standards, were significantly important to ensure the quality and reproducibility of NPs.

The 2024 Nobel Prize in Chemistry was awarded to David Baker, Demis Hassabis, and John M. Jumper for their pioneering work in combining artificial intelligence (AI) and computational chemistry for protein structure prediction, thereby demonstrating the potential for integrating AI and biology. In the field of biomedicine, computational tools are primarily employed for liposome screening, given their stability and extensive research background. Researchers have applied methods such as machine learning and molecular modeling to predict liposome formulations and optimize key parameters, including size, poly disperse index, surface charge, and drug loading efficiency, thereby enhancing the effectiveness and efficiency of their studies [113]. Wang et al. [114] adopted AI and virtual screening to forecast and optimize apparent pKa and delivery efficiency of lipid NPs using a database of nearly 20 million ionizable lipids. After validation in mice, new liposomal molecules that can efficiently deliver mRNA were identified. However, there is an urgent need to develop more advanced AI models for screening and optimizing other engineered NPs, including organic polymer NPs, inorganic NPs, biomimetic NPs, and so on. Furthermore, computational tools offer promising avenues to accelerate the clinical translation of functionalized NPs. AI can, for instance, analyze patients’ data and identify NPs that meet specific patient needs, and virtual patient models can enable preliminary testing of drugs before clinical trials, thereby saving time, resources, and reducing the risk of failure. However, despite the promise of AI, challenges still remain. Building accurate AI models requires a large volumes of high-quality data, with consistent conditions for data collection. Moreover, precise targeting and immunomodulatory properties of NPs are influenced by complex physical, biochemical, and immune network factors, making it more difficulty to develop AI models and increasing development costs.

**Summary**

- Lymph node (LN)-targeted nanoparticles (NPs) boost T-cell activation and remodel the immunosuppressive microenvironment for improved anti-tumor immunity.
- The size, charge, hydrophilicity, deformability, and surface modifications of NPs are critical for efficient LN targeting.
- NPs can regulate suppressive immune cells, reprogram stromal cells, and enhance antigen presentation to optimize immune responses.
- Advanced NP designs and computational tools are pivotal for clinical translation of LN-targeted therapies.

## Abbreviations

FRC: fibroblastic reticular cell
HDL: high-density lipoproteins
HEVs: high endothelial venules
IME: immune microenvironment
LN: lymph node
NPs: nanoparticles
PEG: polyethylene glycol
TDLNs: tumor-draining LNs
